# Coronavirus disease 2019-associated thrombotic microangiopathy treated with plasma exchange and antihypertensive therapy in a patient with HIV: A case report with literature review

**DOI:** 10.1097/MD.0000000000035469

**Published:** 2023-10-13

**Authors:** Eriko Masuda, Kazuaki Fukushima, Yu Hebisawa, Masayuki Tanaka, Akito Ohta, Akifumi Imamura

**Affiliations:** a Department of Infectious Diseases, Tokyo Metropolitan Cancer and Infectious Diseases Center Komagome Hospital, Bunkyo-ku, Tokyo, Japan; b Department of Nephrology, Tokyo Metropolitan Cancer and Infectious Diseases Center Komagome Hospital, Bunkyo-ku, Tokyo, Japan.

**Keywords:** coronavirus disease 2019, malignant hypertension, thrombotic microangiopathy

## Abstract

**Rationale:**

Coronavirus disease 2019 (COVID-19) is an infectious disease that often causes complications in multiple organs and thrombosis due to abnormal blood coagulation. This case report aimed to describe the clinical course of COVID-19-associated thrombotic microangiopathy (TMA) and reviewed the comprehensive information on TMA, thrombotic thrombocytopenic purpura (TTP), and atypical hemolytic uremic syndrome associated with COVID-19 in the past literature.

**Patient concerns:**

A 46-year-old Japanese man was diagnosed with human immunodeficiency virus infection 10 years ago and treated with antiretroviral therapy. The patient presented with fever, malaise, hematuria, and bilateral upper abdominal discomfort for the past 4 days.

**Diagnoses:**

COVID-19-associated TMA was diagnosed based on a positive polymerase chain reaction for severe acute respiratory syndrome coronavirus 2 and laboratory findings such as thrombocytopenia, acute kidney injury, and hemolytic anemia. Malignant hypertension and human immunodeficiency virus infection were also considered as differential diagnoses of TMA.

**Interventions:**

Considering the possibility of TTP, plasma exchange was performed, and glucocorticoids were administered. Hemodialysis was performed for acute kidney injury. Antihypertensive drugs were administered to control the high blood pressure.

**Outcomes:**

Platelet count and renal function improved, and hemodialysis was no longer required. The patient was in good general condition and was discharged from the hospital.

**Lessons:**

COVID-19-associated TMA should be considered as a differential diagnosis during the COVID-19 epidemic. Excessive inflammation and severe COVID-19 are not essential for TMA development. Early intervention using conventional TMA treatments, such as plasma exchange and corticosteroids, might be important in improving prognosis while differentiating between TTP and atypical hemolytic uremic syndrome. Antihypertensive therapy may be helpful in the treatment of COVID-19-associated TMA.

## 1. Introduction

Coronavirus disease 2019 (COVID-19) is an acute infection caused by severe acute respiratory syndrome coronavirus 2 (SARS-CoV-2). Most patients present with mild upper respiratory tract symptoms that resolve spontaneously; however, some are complicated by pneumonia.^[1]^ Some of them further develop acute respiratory distress syndrome, which is a serious condition with acute respiratory failure.^[1,2]^ The pathogenesis of COVID-19 is associated with excessive inflammation (cytokine storm) and blood coagulation abnormalities in and outside the lungs, in addition to direct cellular damage caused by the virus.^[3–6]^ Therapies include antivirals, immunomodulatory agents, and anticoagulants, as required.^[7–9]^ Systemic complications other than those of the lungs are common. Two large observational studies in the United States reported acute kidney injury (AKI) in 32% to 37% of patients hospitalized with COVID-19.^[10,11]^ AKI is associated with the requirement for mechanical ventilation and longer duration of hospitalization.^[10–13]^ Acute tubular necrosis was the predominant kidney pathologic finding in several studies.^[14–17]^ A hypercoagulable state, resulting in arterial and venous thromboses, is common in patients with COVID-19.^[18]^ Conversely, thrombotic microangiopathy (TMA), atypical hemolytic–uremic syndrome (aHUS), and thrombotic thrombocytopenic purpura (TTP) are uncommon complications in patients who develop AKI.^[14,15]^ The pathogenesis of diseases associated with COVID-19 is not well understood, and no optimal treatment has been established. We encountered a case of TMA in a patient with human immunodeficiency virus (HIV) infection that was well-controlled by antiretroviral therapy (ART) based on the findings of AKI, thrombocytopenia, and hemolytic anemia. The patient presented with fever, but no upper respiratory tract symptoms. A positive SARS-CoV-2 PCR test result led to a diagnosis of COVID-19. At the time of admission, he had extreme hypertension with organ damage, particularly AKI, which required hemodialysis. Multidisciplinary treatment with glucocorticoids, plasma exchange (PE), and antihypertensive drugs was initiated; hemodialysis was eventually discontinued, and the patient was discharged from the hospital. We report the patient’s clinical course and possible pathophysiology and review the literature on TMA, aHUS, and TTP as complications of COVID-19. Informed consent was obtained, and the head of the medical team and the institutional review board have responsibility for the anonymization of the patient.

## 2. Case

A 46-year-old Japanese man was diagnosed with an HIV infection 10 years ago. The HIV infection was controlled with fosamprenavir, ritonavir, tenofovir, alafenamide (TAF), and emtricitabine (FTC). Five days before admission, ART was switched to darunavir, cobicistat, TAF, and FTC. Although he had hypertension and was prescribed candesartan, he did not take the drug regularly. Four days prior to admission, he presented to the clinic with fever, fatigue, hematuria, and discomfort in both the upper abdominal areas. He presented with fever but no respiratory symptoms at the time of the visit, and SARS-CoV-2 PCR test was positive. On admission, he had clear consciousness, and his vital signs were temperature of 36.9°C, blood pressure of 228/152 mm Hg, pulse rate of 81/minutes, respiratory rate of 16/minutes, and SpO_2_ of 98% on ambient air. Physical examination revealed dark red blood cell deposits throughout the oral cavity and petechial purpura on the extremities. No eyelid conjunctival pallor was observed. Blood tests revealed severe thrombocytopenia and AKI with platelets of 1000/μL, creatinine of 5.17 mg/dL, and blood urea nitrogen of 79.5 mg/dL (Table 1). In addition to mild anemia, elevated lactate dehydrogenase (LDH) and decreased haptoglobin levels were observed. CD4-positive lymphocyte was 590/μL, and HIV-RNA viral load was undetectable. Computed tomography revealed no obvious pneumonia or intra-abdominal hemorrhage. None of the patient’s family members had renal disease. The patient smoked 1 pack of cigarettes per day from age 20 years to the present, drank 350 ml/day of beer, and had not been vaccinated against SARS-CoV-2.

**Table 1:** Laboratory data on admission.

Complete blood count			Serum chemistries					
WBC	10,700	/μL	Total protein	7.7	g/dL	IgG	1389	mg/dL
RBC	3.81 × 10^4^	/μL	Albumin	4.0	g/dL	IgA	368	mg/dL
Hematocrit	34.2	%	BUN	82	mg/dL	IgM	62	mg/dL
Hemoglobin	12.5	g/dL	Creatinine	5.36	mg/dL	IgE	504.8	IU/mL
MCV	90	fL	Uric acid	11.1	mg/dL	CH50	66.1	CH50/mL
MCHC	36.5	%	Total bilirubin	2.9	mg/dL	C3	133	mg/dL
Platelet count	0.7 × 10^4^	/μL	Direct bilirubin	1.0	mg/dL	C4	32.7	mg/dL
Coagulation studies			AST	113	U/L	Cryoglobulin	Negative	
PT%	96	%	ALT	30	U/L	ANA	40	
PT-INR	1.02		LDH	2831	U/L	RF	<5	IU/mL
APTT	30.1	sec	ALP	54	U/L	MPO-ANCA	<1.0	U/mL
Fibrinogen	519	mg/dL	γ-GTP	72	U/L	PR3-ANCA	<1.0	U/mL
FDP	3.7	μg/mL	Amylase	135	U/L	Glucose	105	mg/dL
D-dimer	1.3	μg/mL	CRP	5.61	mg/dL	HbA1c	5.7	%
AT-III	>120	%	Sodium	135	mEq/L	PRA	147	pg/mL
TAT	3.4	ng/mL	Potassium	2.8	mEq/L	PAC	9.2	ng/mL/h
PIC	1.4	μg/mL	Chloride	95	mEq/L	Free T4	1.30	ng/dL
Factor XIII coagulation activity	143	%	Calcium	8.2	mg/dL	TSH	0.567	ng/dL
ADAMTS13 activity	73	%	Phosphate	4.5	mg/dL	NT-proBNP	19213	pg/mL
Urinalysis			Creatine kinase	2812	U/L	Infectious disease		
Urine specific gravity	1.012		Haptoglobin	<10	mg/dL	HBsAg	Negative	
pH	6.0		Direct coombs test	negative		HBV-DNA	Negative	
Protein	2+		Indirect coombs test	negative		HCV-Ab	Negative	
Sugar	(−)		Iron	123	μg/dL	RPR	Negative	
Occult blood	3+		UIBC	178	μg/dL	HIV-RNA	Negative	
Protein	151	mg/dL	TIBC	301	μg/dL	CD4-positive lymphocyte	590	/μL
Urine total protein/g•Cr	2.08	g/g•Cr						
β2-microglobulin	57407	μg/L						
Myoglobin	10900	ng/mL						
α1-microglobulin	153.3	mg/L						
N-acetyl-β-D-glucosaminidase	29.7	U/L						

ALP = alkaline phosphatase, ALT = alanine transaminase, ANA = antinuclear antibody, APTT = activated partial thromboplastin time, AST = aspartate aminotransferase, AT-III = antithrombin III, BUN = blood urea nitrogen, CH50 = 50% hemolytic complement activity, FDP = fibrinogen/fibrin degradation products, HbA1c = hemoglobin A1c, HBsAg = hepatitis B virus antigen, HBV-DNA = hepatitis B-deoxyribonucleic acid, HCV-Ab = hepatitis C-antibody, HIV-RNA = human immunodeficiency virus-ribonucleic acid, LDH = lactate dehydrogenase, MCHC = mean corpuscular hemoglobin concentration, MCV = mean corpuscular volume, MPO-ANCA = myeloperoxidase-antineutrophil cytoplasmic antibody, NT-proBNP = N-terminal pro-brain natriuretic peptide, PAC = plasma aldosterone concentration, PIC = alpha2-plasmin inhibitor-plasmin complex, PR3-ANCA = proteinase-3-antineutrophil cytoplasmic antibodies, PRA = plasma renin activity, PT = prothrombin time, PT-INR = prothrombin time-international normalized ratio, RBC = red blood cell, RF = rheumatoid factor, RPR = rapid plasma reagin test, TAT = thrombin-antithrombin complex, TIBC = total iron binding capacity, TSH = thyroid stimulating hormone, UIBC = unsaturated iron binding capacity, WBC = white blood cell, γ-GTP = γ-glutamyl transpeptidase.

**Figure 1.**
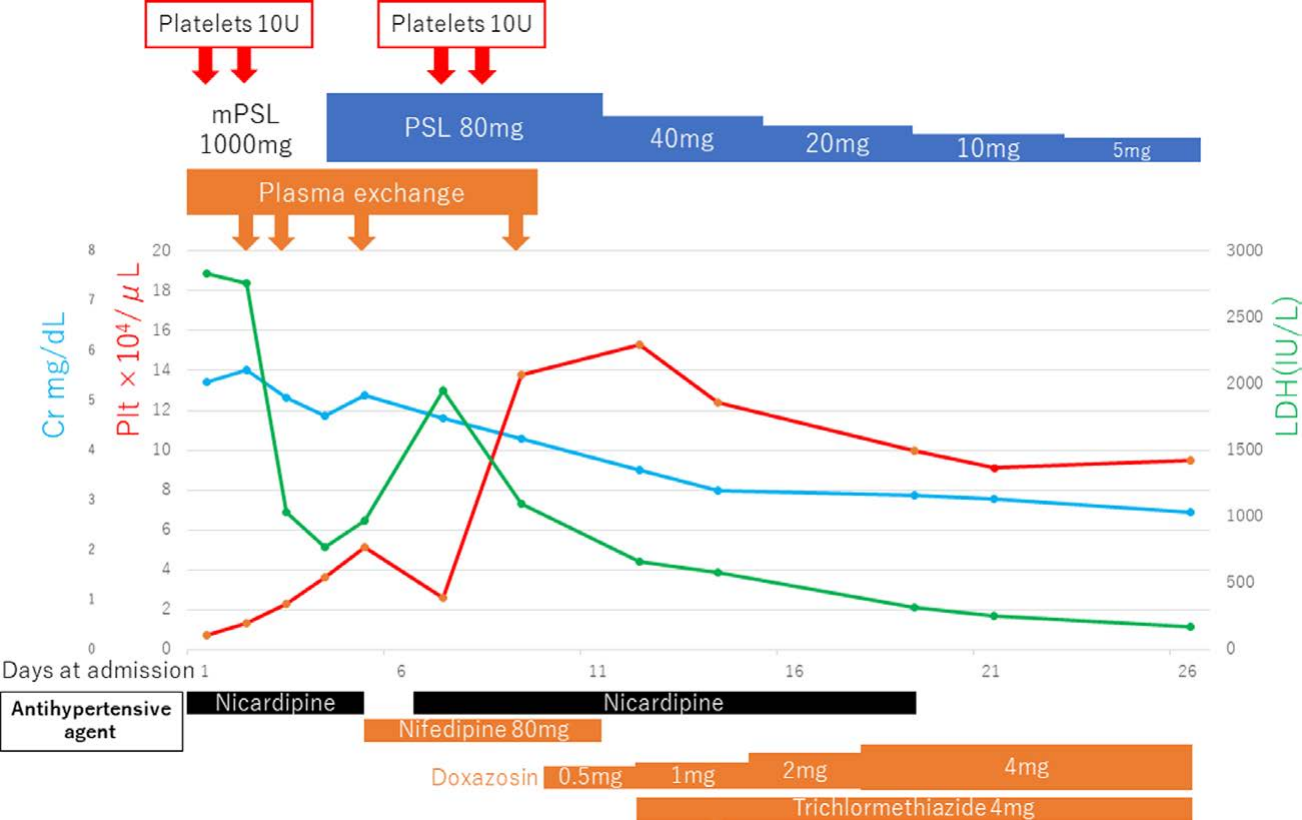
Clinical course during hospitalization.

The patient was considered to have TMA because of low platelet count, AKI, low haptoglobin, and high LDH level. Prothrombin time, activated partial thromboplastin time, fibrinogen level, and fibrin/fibrin degradation products were normal, and disseminated intravascular coagulation was excluded. PLASMIC score^[19]^ was 5 points. Considering the possibility of TTP, steroid pulse therapy (methylprednisolone 1000 mg for 3 days) was initiated on the second day of the disease, followed by prednisolone 1 mg/kg/day (Fig. 1). PE and hemodialysis were initiated on the same day. Continuous intravenous nicardipine was initiated to markedly elevate the blood pressure. As he had no central nervous system symptoms, twenty units of platelets were transfused on the first and second days. As he did not show pneumonia or oxygen demand, the patient was judged to have mild COVID-19 based on the World Health Organization’s severity classification,^[20]^ and antiviral treatments for SARS-CoV-2 were not initiated.

On the 5th day, platelets increased to 51,000/μL after 2 plasma exchanges. ADAMTS13 activity was 73%, showing no decrease, and TTP was ruled out. Glucocorticoids were tapered and discontinued. Systolic blood pressure dropped below 180 mmHg, and nicardipine administration was terminated on the 6th day. However, on the day after termination, the systolic blood pressure rose again to 200 mm Hg. Blood tests showed a decrease in platelet count to 26,000/μL and an increase in LDH level. Twenty units of platelets were transfused over 2 days. Intravenous nicardipine was resumed to maintain systolic blood pressure below 140 mm Hg. Blood pressure was well-controlled with oral nifedipine (80 mg), doxazosin, and trichloromethiazide. On the ninth day, platelets increased to 138,000/μL. As the blood pressure stabilized at approximately 130 mm Hg, intravenous nicardipine was terminated. Renal function gradually improved, and on the 19th day, creatinine decreased from 5.6 mg/dL (first day) to 3.06 mg/dL, and urinary findings also improved; therefore, hemodialysis was terminated. ART was resumed with raltegravir, TAF, and FTC. His general condition stabilized and was discharged on the 28th day. The patient was under outpatient observation, and no similar episodes were observed for up to 8 months after discharge.

## 3. Discussion

Here, we report the case of a patient with a well-controlled HIV infection who was diagnosed with simultaneous COVID-19 and TMA based on the findings of AKI, thrombocytopenia, and hemolytic anemia. The multidisciplinary treatment for TMA includes glucocorticoids, PE, hemodialysis, and antihypertensive medications for extreme hypertension. The patient was discontinued from dialysis and his renal function recovered.

A literature review was conducted to investigate the relationship between COVID-19 and TMA using the PubMed database. The search terms were (“thrombotic microangiopathy” OR “TMA” OR “thrombotic thrombocytopenic purpura” OR “TTP” OR “Atypical hemolytic uremic syndrome” OR “aHUS”) AND (“COVID-19” OR “SARS-CoV-2”). Patients with suspected TMA due to vaccination were excluded from the study. A total of 63 patients were identified as having TMA associated with COVID-19. Of these, 24 were reported as COVID-19-related TMA (Table 2)^[21–39]^, 23 as TTP (Table 3)^[40–61]^, and 16 as aHUS (Table 4).^[62–73]^

**Table 2:** COVID-19-related Thrombocytopenic microangiopathy.

Author	Age	Sex	Underlying illnesses/past history	COVID-19 to TMA Diagnosis	ADAMTS13	HD	PE	Steroids	RTX	Eculizumab	Caplacizumab	Outcome
Masuda, et al (2023)	46	M	HIV, HTN	Concurrent	Not low	+	+	−	−	−	−	Recovered
Malgaj Vrecko et al (2022)^[21]^	32	M	Not mentioned	Not mentioned	Not low	−	+	+	−	−	−	Recovered
Fahim et al (2022)^[22]^	45	F	Kidney transplant, HTN, obesity, normocytic anemia, sleeve gastrectomy, obstructive sleep apnea, gestational diabetes, and preeclampsia	Concurrent	NA	−	−	−	−	−	−	Partial recovery
Pinte et al (2022)^[23]^	23	M	HTN	Concurrent	not low	+	−	−	−	−	−	Partial recovery
Maramattom et al (2022)^[24]^	55	F	Not mentioned	16	NA	+	+	+	−	−	−	Partial recovery
López-Marín et al (2022)^[25]^	78	M	HTN, ischemic cardiopathy, COPD	3	NA	+	+	+	−	−	−	Not mentioned
Gandhi et al (2022)^[26]^	27	M	HTN	11	Not low	+	−	+	−	+	−	Partial recovery
Tarasewicz et al (2021)^[27]^	41	M	Kidney transplant (immunoglobulin (Ig) A nephropathy)	30	Not low	+	+	−	−	−	−	Retransplantation
Elkayam et al (2021)^[28]^	44	F	Not mentioned	Concurrent	Not low	+	+	−	−	−	−	Recovered
El Sissy et al (2021)^[29]^	66	M	Not mentioned	Concurrent	Not low	+	−	−	−	−	−	Partial recovery
El Sissy et al (2021)	71	M	Kidney transplant	12	Not low	−	+	−	−	+	−	Recovered
El Sissy et al (2021)	35	M	Not mentioned	30	Not low	+	+	−	−	+	−	Partial recovery
El Sissy et al (2021)	26	F	Kidney transplant	Concurrent	Not low	+	+	−	+	+	−	Partial recovery
El Sissy et al (2021)	38	F	Kidney transplant	10	Not low	−	−	−	−	−	−	Recovered
Kulkarni et al (2021)^[30]^	25	F	Not mentioned	7	Not low	+	−	+	−	−	−	Recovered
Utebay et al (2021)^[31]^	76	M	Af	7	Not low	−	+	+	−	+	−	Recovered
Dashti-Khavidaki et al (2021)^[32]^	39	F	Liver transplant, liver cirrhosis due to primary sclerosing cholangitis	9	Not low	−	+	−	−	−	−	Recovered
Boudhabhay et al (2021)^[33]^	46	M	HTN, obesity		Not low	+	−	−	−	+	−	Recovered
Jespersen Nizamic et al (2021)^[34]^	49	F	Kidney transplant (CKD secondary to focal segmental glomerulosclerosis)	12	NA	−	−	−	−	−	−	Recovered
Safak et al (2021)^[35]^	34	M	HTN	Concurrent	NA	−	−	−	−	+	−	Recovered
Sharma et al (2021)^[36]^	63	F	Metastatic Cholangiocarcinoma (received chemotherapy with gemcitabine)	Concurrent	Not low	+	+	+	−	−	−	Recovered
Bascuñana et al (2021)^[37]^	40	M	Kidney transplant (kidney failure secondary to Liddle syndrome)	Concurrent	Not low	+	+	+	−	−	−	Recovered
Airoldi et al (2020)^[38]^	56	M	HCV	Concurrent	NA	−	−	+	−	−	−	Recovered
Jhaveri et al (2020)^[39]^	69	F	Asthma	Concurrent	Not low	+	−	−	−	+	−	Died

Af = atrial fibrillation, CKD = chronic kidney disease, COPD = chronic obstructive pulmonary disease, COVID-19 = coronavirus disease 2019, F = female, HCV = hepatitis C, HD = hemodialysis, HIV = human immunodeficiency virus, HTN = hypertension, M = male, NA = not assessed, PE = plasma exchange, RTX = rituximab, TMA = thrombotic microangiopathy.

**Table 3:** COVID-19-related thrombotic thrombocytopenic purpura

Author	Age	Sex	Underlying illnesses/past history	COVID-19 to TMA Diagnosis	ADAMTS13	HD	PE	Steroids	RTX	eculizumab	caplacizumab	Outcome
Chaudhary et al (2022)^[40]^	33	F	Not mentioned	Concurrent	Low (inhibitor +)	−	+	+	+	−	+	Recovered
Mandyam et al (2022)^[41]^	26	M	Not mentioned	21	Low (inhibitor +)	+	+	+	+	−	+	Recovered
Desai et al (2022)^[42]^	40	F	Not mentioned	Concurrent	Low	−	+	+	+	−	+	Recovered
Kesavan et al (2022)^[43]^	54	F	Not mentioned	Concurrent	NA	+	−	+	−	−	−	Died
Dhingra et al (2021)^[44]^	35	F	Not mentioned	Concurrent	Low	−	+	+	+	−	−	Recovered
Nicolotti et al (2021)^[45]^	44	F	obesity, DVT	Concurrent	Low (inhibitor +)	−	+	+	+	−	+	Recovered
Shankar et al (2021)^[46]^	30	M	obesity	7	Low	−	+	+	−	−	+	Recovered
Prasad Verma et al (2021)^[47]^	21	M	Not mentioned	Concurrent	NA	−	−	+	−	−	−	Died
Tehrani et al (2021)^[48]^	25	F	pregnancy	Concurrent	Low (inhibitor +)	−	+	+	−	−	−	Recovered
Darnahal et al (2021)^[49]^	56	F	breast cancer (locally advanced)	Concurrent	Low (inhibitor +)	−	+	+	+	−	−	Died
Tehrani et al (2021)^[50]^	57	F	Not mentioned	Concurrent	Not Low (inhibitor +?)	−	−	+	−	−	−	Recovered
Tehrani et al (2021)	38	M	Not mentioned	Concurrent	Low (inhibitor +)	−	+	+	+	−	−	Recovered
Maharaj et al (2021)^[51]^	69	F	TTP, cerebral infarct	Concurrent	Low	−	+	+	−	−	−	Died
Beaulieu et al (2021)^[52]^	70	M	peripheral artery disease, dyslipidemia	19	Low (inhibitor +)	−	+	+	−	−	−	Recovered
Law et al (2021)^[53]^	47	F	Not mentioned	Concurrent	Low (inhibitor +)	−	+	+	+	−	−	Recovered
Aminimoghaddam et al (2021)^[54]^	21	F	pregnancy	Concurrent	NA	+	+	+	−	−	−	Recovered
Alhomoud et al (2021)^[55]^	62	M	TTP, Crohn disease, G6PD deficiency	Concurrent	Low (inhibitor +)	−	+	+	+	−	−	Recovered
Cohen et al (2021)^[56]^	62	F	SLE, APS, CVA, hyperlipidemia trigeminal neuralgia, and hypothyroidism	Concurrent	Low (inhibitor +)	−	+	+	+	−	+	Recovered
Altowyan et al (2020)^[57]^	39	M	DM, HTN	Concurrent	NA	−	+	+	+	−	−	Recovered
Dorooshi et al (2020)^[58]^	81	F	HTN	Concurrent	NA	−	−	+	−	−	−	Died
Hindilerden et al (2020)^[59]^	74	F	HTN	Concurrent	Low (inhibitor +)	−	+	+	−	−	−	Recovered
Capecchi et al (2020)^[60]^	55	F	TTP	30	Low (inhibitor +)	−	+	+	−	−	+	Recovered
Albiol et al (2020)^[61]^	57	F	HTN, breast cancer	Concurrent	Low (inhibitor +)	−	+	+	−	−	−	Recovered

APS = antiphospholipid syndrome, COVID-19 = coronavirus disease 2019, CVA = cerebral vascular accident, DM = diabetes mellitus, DVT = deep vein thrombosis, F = female, G6PD = glucose 6 phosphate dehydrogenase, HD = hemodialysis, M = male, NA = not assessed, PE = plasma exchange, RTX = rituximab, SLE = systemic lupus erythematosus, TMA = thrombotic microangiopathy, TTP = thrombotic thrombocytopenic purpura.

**Table 4:** COVID-19-related atypical hemolytic uremia syndrome.

Author	Age	Sex	Underlying illnesses/past history	COVID-19 to TMA diagnosis	ADAMTS13	HD	PE	Steroids	RTX	Eculizumab	Caplacizumab	Outcome
Smarz-Widelska et al (2022)^[62]^	19	M	aHUS	Concurrent	NA	−	−	−	−	+	−	Recovered
Smarz-Widelska et al (2022)	23	M	aHUS	Concurrent	NA	−	−	−	−	+	−	Recovered
Boldig et al (2022)^[63]^	36	F	aHUS	Concurrent	NA	+	−	+	−	+	−	Died
Tiwari et al (2022)^[64]^	26	M	Not mentioned	Concurrent	NA	+	+	−	−	−	−	Partial recovery
Leone et al (2022)^[65]^	77	F	HTN, RA, HBV	13	not low	+	−	+	−	+	−	Partial recovery
Leone et al (2022)	79	F	Heart disease, initial cerebral vasculopathy, CKD	28	Not low	+	−	+	−	+	−	Partial recovery
Tatar et al (2022)^[66]^	29	F	aHUS	Concurrent	NA	−	−	+	−	+	−	Recovered
Korotchaeva et al (2022)^[67]^	49	F	Not mentioned	Concurrent	Not low	+	−	−	−	+	−	Recovered
Korotchaeva et al (2022)	39	M	HTN	10	Not low	+	+	+	−	+	−	Partial recovery
Korotchaeva et al (2022)	66	F	DM	Concurrent	Not low	−	−	−	−	+	−	Died
Gill et al (2022)^[68]^	32	M	Childhood leukemia, heart transplant	Concurrent	Not low	+	+	+	−	+	−	Recovered
Tarasewicz et al (2021)^[69]^	41	M	Kidney transplant (immunoglobulin (Ig) A nephropathy)	30	Not low	+	+	−	−	−	−	Retransplantation
Kurian et al (2021)^[70]^	25	M	aHUS	Concurrent	not low	−	+	+	−	+	−	Recovered
Kurian et al (2021)^[70]^	31	F	Not mentioned	Concurrent	not low	+	+	+	−	+	−	Recovered
Mat et al (2021)^[71]^	39	M	HTN, CKD, low-grade esophagitis	Concurrent	Not low	+	+	+	-	+	-	Partial recovery
Ville et al (2021)^[72]^	28	F	aHUS	Concurrent	NA	-	-	-	-	+	-	Recovered
Trimarchi et al (2020)^[73]^	24	M	aHUS, kidney transplant	Concurrent	NA	-	-	+	-	+	-	Recovered

aHUS = atypical hemolytic uremic syndrome, CKD = chronic kidney disease, COVID-19 = coronavirus disease 2019, DM = diabetes, F = female, HBV = hepatitis B, HD = hemodialysis, HTN = hypertension, M = male, NA = not assessed, PE = plasma exchange, RA = rheumatoid arthritis, RTX = rituximab, TMA = thrombotic microangiopathy.

The cases of COVID-19-related TMA had a male predominance (58.3%) with a mean age of 44.5 years (interquartile range: 34.75–57.75 years). Seven post-renal transplant patients and 1 post-liver transplant were included in this study. None of the patients had a history of TMA, and 7 had a history of hypertension. Fever (n = 9, 37.5%), upper respiratory symptoms (n = 6, 25.0%), shortness of breath (n = 5, 20.8%), and hematuria (n = 3, 12.5%) were the most common subjective symptoms. None of the included cases (n = 18) reported an ADAMTS13 activity of <10%. Renal biopsies performed in 15 patients revealed microthrombosis and tubular damage, typical of TMA. Fourteen patients (63.6%) were diagnosed with TMA within 7 days of the onset of COVID-19. Twelve (50.0%) patients underwent PE. Nine (37.5%) patients received glucocorticoids. One patient received rituximab, and 8 received eculizumab. More than half of the patients (n = 15, 62.5%) underwent hemodialysis, and 7 could not undergo dialysis. Only 1 (4.2%) patient died; a 69-year-old woman with preexisting asthma received eculizumab and underwent hemodialysis.

The cases of COVID-19-related TTP had a female predominance (69.6%), with a mean age of 46.8 years. Three of the 23 patients had a history of TTP. All included patients (n = 18, 78.2%) had a reported ADAMTS13 activity of < 10%. None of the patients underwent renal biopsy. The majority (n = 19, 82.6%) of patients were diagnosed with TTP at the onset of COVID-19. Nineteen patients (82.6%) underwent plasma exchange, and all patients received glucocorticoids. Eleven patients (45.8%) received rituximab, 7 (29.2%) received caplacizumab, and 5 (20.8%) received intravenous immunoglobulin. Three patients (13.0%) underwent hemodialysis. Five patients (21.7%) died, with death more frequently in the TMA cases.

Sixteen patients with aHUS with a mean age of 46.8 years, half males and half females were reported. Nearly half of the patients (n = 7; 43.8%) had a history of aHUS. Six patients (37.5%) harbored genetic mutations. Six patients (37.5%) underwent renal biopsy. Six (37.5%) patients underwent PE. Ten patients (62.5%) received glucocorticoids, and 14 patients (87.5%) received eculizumab. Nine patients (56.3%) received hemodialysis, and 3 patients could not discontinue dialysis. Two cases died.

Our patient presented with thrombocytopenia, AKI, and hemolytic anemia (elevated LDH and low haptoglobin levels), suggesting TTP, aHUS, or TMA. TTP was ruled out because ADAMTS13 activity was normal. He had no history of medications that could induce TMA. Shiga toxin-producing Escherichia coli associated HUS was ruled out because no Shiga toxin was detected in the stool, and diarrhea symptoms were absent. aHUS may be caused by inherited pathogenic mutations in complement genes or autoantibodies directed against complement proteins. Although genetic testing and antibodies against complement factors are required to diagnose aHUS, these tests were not performed because the complement function results were normal in this case.

We discuss the possible causes of secondary TMA. Malignant hypertension is a well-known cause of TMA. In our patient, hypertension was noted prior to admission, and his systolic blood pressure was approximately 150 mmHg. A history of hypertension, high mean arterial pressure, significant renal impairment, modest thrombocytopenia, and lack of severe ADAMTS13 deficiency (activity < 10%) at diagnosis are clues for diagnosing malignant hypertension-induced TMA.^[74,75]^ Several patients have been reported to be in remission with blood pressure control alone without PE.^[76–78]^ In this case, malignant hypertension-induced TMA was considered; however, severe thrombocytopenia was observed and atypical. Several previous reports on COVID-19-related TMA have described patients with a history of hypertension.^[23,25,26,33,35]^ Therefore, concluding that hypertension alone induced TMA is difficult, and we concurrently administered PE and glucocorticoids. Conversely, the fact that the patient’s condition improved with appropriate antihypertensive therapy suggests that extreme hypertension may have exacerbated the disease. TMA associated with HIV infection was less likely to be involved in this case because 10 years had passed since the onset of the HIV infection, and the patient had good compliance. The anti-HIV drugs were unlikely related to TMA because it required only a short time for symptoms to appear after changing the drugs, and the drugs were not reported to cause drug-induced TMA. The lack of improvement in renal impairment and requirement for several antihypertensive drugs prompted us to use raltegravir and TAF/FTC as anti-HIV drugs. Based on these findings, TMA secondary to COVID-19 was diagnosed. In this case, TMA was caused by the absence of COVID-19 symptoms, such as upper respiratory symptoms. Secondary TMA generally develops in parallel with the activity of the primary disease and may not occur in TMA secondary to COVID-19.

PE, glucocorticoids, hemodialysis, and blood pressure control contributed to improvements in renal function and thrombocytopenia. PE has been previously reported to be effective. The importance of blood pressure control has been suggested because the disease is exacerbated when blood pressure control is inadequate. He developed AKI requiring hemodialysis but responded well to treatment. Dialysis was discontinued, and renal function returned to baseline.

COVID-19-related TMA should be considered as a differential diagnosis during the COVID-19 epidemic. Excessive inflammation and severe COVID-19 are not essential for TMA development. Early intervention with conventional TMA treatments, such as PE and corticosteroids, might be important in improving prognosis while differentiating between TTP and aHUS. Antihypertensive therapy may be helpful in the treatment of COVID-19-associated TMA.

## Acknowledgments

We thank all the staff at the Tokyo Metropolitan Cancer and Infectious Diseases Center, Komagome Hospital, for their excellent patient care.

## Author contributions

**Conceptualization:** Eriko Masuda, Kazuaki Fukushima.

**Supervision:** Kazuaki Fukushima.

**Writing – original draft:** Eriko Masuda.

**Writing – review & editing:** Eriko Masuda, Kazuaki Fukushima, Yu Hebisawa, Masayuki Tanaka, Akito Ohta, Akifumi Imamura.
